# A DTW-Based Spatio-Temporal Synchronization Method for Radar and Camera Fusion

**DOI:** 10.3390/s26072108

**Published:** 2026-03-28

**Authors:** Jingjing Li, Juan Liu, Xiuping Li, Chengliang Zhong, Xiyan Sun

**Affiliations:** 1 Guangxi Key Laboratory of Precision Navigation Technology and Application, Guilin University of Electronic Technology, Guilin 541004, China; lijingjing@guet.edu.cn (J.L.); liujuan@mails.guet.edu.cn (J.L.); lxp863@gmail.com (X.L.); chengliabgzhong@mails.guet.edu.cn (C.Z.); 2 Information and Communication School, Guilin University of Electronic Technology, Guilin 541004, China; 3 National & Local Joint Engineering Research Center of Satellite Navigation Positioning and Location Service, Guilin 541004, China; 4 GUET-Nanning E-Tech Research Institute Co., Ltd., Nanning 530031, China

**Keywords:** spatio-temporal synchronization, DTW, radar and camera fusion

## Abstract

Roadside perception systems, also known as roadside units (RSUs), are critical in Vehicle-to-Everything (V2X) applications, yet spatio-temporal asynchrony between multiple sensors severely compromises the accuracy of fusion. In this paper, a spatio-temporal synchronization method for millimeter-wave (MMW) radar and camera fusion is proposed, integrating target matching based on dynamic time warping (DTW) with spatio-temporal parameter estimation. Leveraging the advantages of DTW in time-series alignment to calculate the similarity between radar and visual trajectories enables target matching and parameter estimation in sparse scenes. This method was validated on a real-world dataset containing over 30 pedestrian trajectories, covering scenarios with varying densities ranging from one to six pedestrians. The results indicate a temporal offset of 0.116 s between the camera and radar. Following synchronization, the average spatial deviation decreased from 1.4358 to 0.1074 m in the x-direction (i.e., across the road) and from 3.0732 to 0.1775 m in the y-direction (i.e., along the road). Consequently, this method provides an efficient solution for deploying roadside perception systems in sparse traffic environments.

## 1. Introduction

The evolution of Intelligent Transportation Systems (ITSs) [1] over the past decade has significantly promoted the deployment of RSUs. As a core component of Cooperative Vehicle–Infrastructure Systems (CVISs), roadside units extend the perception range beyond the line of sight of onboard sensors [2]. Among various sensor configurations, the combination of cameras and MMW radar is gaining increasing popularity. Cameras provide rich semantic information and high angular resolution, while MMW radar offers robust ranging and velocity measurement capabilities that are independent of lighting conditions [3,4,5]. The fusion of such heterogeneous sensors is crucial for achieving all-weather and comprehensive traffic monitoring.

However, roadside sensors typically operate within independent subsystems with inconsistent sampling frequencies and misaligned internal clocks. Furthermore, their spatial coordinate systems are physically separated and lack a common reference benchmark [6]. Without precise calibration in the temporal and spatial dimensions, data association becomes ambiguous, thereby compromising the accuracy of subsequent data fusion.

Existing inter-sensor calibration methods are primarily categorized into target-based and target-less approaches. Target-based calibration utilizes dedicated targets with specific geometric features, such as checkerboards [7,8,9] or corner reflectors [10,11], for offline calibration. However, this approach requires cumbersome on-site operations and traffic disruption, making it ill-suited for large-scale deployment. To address this issue, recent studies have explored target-less calibration methods. For example, Yuan et al. proposed a novel and reliable depth-continuous edge extraction algorithm that leads to more accurate calibration parameters [12]; Cheng et al. utilized deep learning techniques to extract common features between radar and camera systems, developing an online target-less calibration [13]; Ou et al. proposed a novel target-less Lidar–camera calibration method based on cross-modality structure consistency and ensured global convergence within a large search range [14]. Additionally, Du et al. proposed extracting the time headway of continuous vehicular flow using “virtual detection lines” to match cross-sensor trajectories [15]. While this method proves effective in highway scenarios with dense and continuous traffic, the scarcity of targets in sparse traffic environments leads to the degradation of statistical features. Consequently, such methods are prone to matching failures and parameter divergence.

Therefore, this paper proposes a spatio-temporal synchronization method tailored for sparse traffic conditions. The main contributions of this paper are summarized as follows:

(1) We propose a trajectory-matching strategy based on DTW and the Hungarian algorithm. The calculated DTW distance serves as the cost matrix for the Hungarian algorithm to achieve multi-target matching.

(2) We construct a linear regression optimization model. By utilizing the time series of trajectories aligned via DTW and applying the least squares method, the model achieves a joint estimation of the temporal offset and spatial deviation.

(3) The proposed method is validated using pedestrian data. Comparisons of matching results and spatio-temporal deviations before and after synchronization demonstrate that the method achieves effective synchronization, even in scenarios with sparse traffic flow.

## 2. Related Work

### 2.1. Target-Based Calibration

Target-based calibration methods typically utilize specific calibration targets to establish correspondence between the coordinate systems of radar and camera sensors. For instance, Cheng et al. proposed an extrinsic calibration method for 3D radar and cameras in which a corner reflector was placed on the ground and data from both sensors were captured simultaneously via ROS. Radar–camera correspondences were then established based on timestamps and subsequently employed to solve the Perspective-n-Point (PnP) problem, thereby obtaining the extrinsic calibration matrix [16]. Domhof et al. adopted a single-target calibration design and developed an open-source tool integrated into the Robot Operating System (ROS) to facilitate its implementation [17]. Song et al. utilized an AR marker detectable by radar to simultaneously measure the marker’s position relative to the camera and radar coordinate systems; by applying paired point registration, they obtained the transformation between the radar and camera coordinate systems [18].

### 2.2. Target-Less Calibration

In contrast, target-less calibration does not rely on specific calibration tools. He et al. proposed a novel spatial calibration method that obtains corresponding 2D–3D point pairs by combining tracking results from radar and camera sensors. Initial extrinsic parameters are estimated via the PnP algorithm and subsequently refined through nonlinear optimization to enhance the calibration accuracy [19]. Durmaz et al. introduced a fully target-less calibration framework that estimates the rigid spatial transformation between radar and camera coordinate frames by aligning the trajectories of moving objects observed by both sensors [20]. Furthermore, Hu et al. proposed a general calibration framework based on the Iterative Best Match (IBM) algorithm. This method improves alignment by optimizing correspondences between sensors, thereby eliminating the need for traditional point-to-point matching and predefined calibration targets [21]. Liu et al. proposed a track association algorithm for heterogeneous sensors to achieve target-less calibration between the radar and camera. Corresponding points were extracted from millimeter-wave radar and image coordinate systems, and external parameters were obtained by applying PnP and nonlinear optimization algorithms [22]. Furthermore, Schöller et al. proposed the first data-driven method for automatic rotational radar–camera calibration without dedicated calibration targets. They employed a boosting-inspired training algorithm, where they trained the fine network on the residual error of the coarse network [23].

### 2.3. Dynamic Time Warping

H. Sakoe and S. Chiba pioneered the application of dynamic time warping to the field of speech recognition [24]. Subsequently, other researchers have continuously refined the algorithms and applied them across various fields. Chen et al. proposed a methodology for the joint simulation of the dynamic RCS under two DOF models and the subsequent comparison and analysis of the resulting sequence data via the dynamic time warping algorithm [25]. Mehta, R et al. utilized DTW as a classical baseline for temporal alignment to demonstrate the superior performance of deep learning architectures in recognizing continuous human activities from radar micro-Doppler signatures [26]. To address the temporal variations in gesture execution, Liu et al. utilized DTW to synchronize radar and vision sequences, ensuring robust feature alignment across different sensing modalities [27].

## 3. Methodology

### 3.1. System Framework

**Figure 1:**
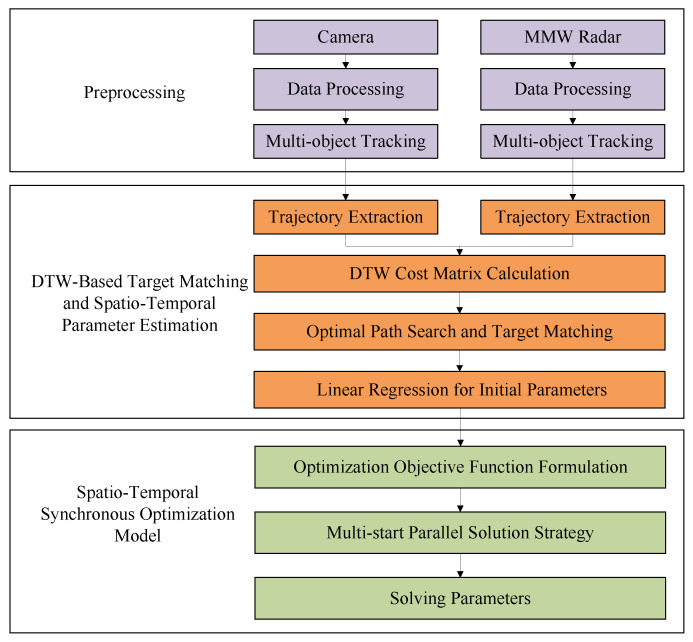
Framework of the spatio-temporal synchronization method based on radar–camera fusion.

The framework of the proposed spatio-temporal synchronization method based on radar–camera fusion is illustrated in Figure 1. The framework primarily consists of three modules: the preprocessing module, the DTW-based target matching and spatio-temporal parameter estimation module, and the spatio-temporal synchronous optimization model.

First, the preprocessing module performs coordinate mapping and interpolation on independently acquired visual and radar data, generating two sets of independent trajectory sequences using a multi-object tracking algorithm. Subsequently, the DTW-based spatio-temporal parameter estimation module calculates similarity cost matrices using global trajectory shapes. Combined with the Hungarian algorithm, it enables multi-object matching in sparse scenes and obtains initial spatio-temporal offset parameters through linear regression. Finally, the initial parameters are input into the spatio-temporal synchronization optimization model to output precise spatio-temporal synchronization parameters.

### 3.2. Preprocessing

#### 3.2.1. Data Acquisition and Processing

Continuous data streams of pedestrians were synchronously collected from the camera and MMW radar to facilitate subsequent processing. Our proposed method does not interfere with traffic flow and it only requires naturally behaving pedestrians to pass by during data collection.

The camera recorded video at a frame rate of 30 fps, utilizing an object detection algorithm to generate bounding boxes for targets on a frame-by-frame basis. The bottom center was selected as the anchor point within each bounding box, and its pixel coordinates were extracted to characterize the target’s position on the image plane. Four pairs of ground control points were selected to calculate the homography matrix using the Direct Linear Transformation (DLT) method [28], followed by the preliminary calibration of pixel coordinates to convert them into world coordinates.

The MMW radar output is structured detection data at a frequency of 30 Hz, including the 2D position, velocity, and ID of the targets. The radar coordinate system was established as a 2D Cartesian coordinate system, with the installation center of the radar sensor serving as the origin. Specifically, the Y-axis was defined as the longitudinal direction extending along the road, while the X-axis was defined as the lateral direction perpendicular to the road alignment.

Although the nominal sampling frequencies of the camera and MMW radar were the same, non-uniform sampling phenomena inevitably occurred in the sensor data streams during actual acquisition due to constraints in system scheduling and data transmission latencies [29]. To establish a unified temporal baseline, the camera’s timestamp sequence was selected as the reference, and radar trajectories were resampled to align with the camera timestamps using linear interpolation. Despite the highly non-rigid and stochastic nature of pedestrian motion, their state of motion can be approximated as uniform linear motion within the extremely short sampling intervals of the sensors [30]. Additionally, according to the Taylor series truncation error bound, the maximum displacement error $E_{max}$ induced by linear interpolation is governed by the maximum acceleration $a_{max}$, expressed as $E_{max}\leq \frac{1}{8}a_{max}{\left(\Delta t\right)}^{2}$. Even assuming an extreme pedestrian maneuvering acceleration of $a_{max}=10$ m/s^2^, the theoretical maximum interpolation error is approximately 1.36 mm. This sub-millimeter error is orders of magnitude smaller than the intrinsic spatial noise of commercial MMW radars. Thus, the linear interpolation introduces negligible systematic error and perfectly preserves the trajectory fidelity for subsequent DTW alignment.

#### 3.2.2. Multi-Target Tracking

To achieve data synchronization and matching between the camera and millimeter-wave radar, extracting stable and continuous motion trajectories from raw detection data is essential. In this paper, a multi-object tracking algorithm based on Kalman filtering is employed to accomplish this preprocessing task. First, detection data captured at time t are matched with the predicted states from the previous timestamp using the Global Nearest Neighbor (GNN) data association method. For successfully associated observations, a Kalman filter is utilized to update the state variables of the trajectory and predict the motion state for the subsequent timestamp [31]. Conversely, unmatched observations are directed into a buffer for aggregation analysis via the DBSCAN clustering algorithm [15]. Specifically, the clustering parameters were set as follows: the neighborhood radius ε = 0.6 m was selected to align with the typical physical width of a pedestrian, and the minimum number of points was set to MinPts = 3 to accommodate the sparse point cloud returns from low-RCS targets. When a cluster persists and accumulates data points for a duration exceeding a preset threshold of 10 consecutive frames, it is promoted to a valid new trajectory and assigned a unique identifier. For trajectories that temporarily fail association, the number of consecutive missing frames is recorded; tracking is resumed if re-association occurs within a specified time window. Otherwise, the target is considered to have disappeared, and the trajectory is terminated.

### 3.3. DTW-Based Spatio-Temporal Parameter Estimation

#### 3.3.1. DTW-Based Trajectory Matching

DTW utilizes the fundamental concepts of dynamic programming to identify an optimal alignment path between two time series, minimizing the cumulative distance between corresponding data points [32,33].

The algorithm initially extracts the overlapping segments along the road direction for each camera–radar trajectory pair. Subsequent processing is performed only if the length of this overlap exceeds a preset threshold $L_{min}$. Let the *i*-th camera trajectory be denoted as $C_{i}=\left\{\left(x_{q}^{c},y_{q}^{c},t_{q}^{c}\right)|q=1,2,\ldots ,m\right\}$, where *q* serves as the data point index and *m* represents the total number of frames recorded for this specific camera trajectory. Similarly, let the *j*-th radar trajectory be denoted as $R_{j}=\left\{\left(x_{l}^{r},y_{l}^{r},t_{l}^{r}\right)|l=1,2,\ldots ,n\right\}$, with *l* denoting the index and *n* indicating the total number of radar data points. The variables *x*, *y*, and *t* represent the lateral coordinate (across the road), longitudinal coordinate (along the road), and timestamp, respectively. Furthermore, let $Y_{C_{i}}=\left\{y_{1}^{c},\ldots ,y_{m}^{c}\right\}$ and $Y_{R_{j}}=\left\{y_{1}^{r},\ldots ,y_{n}^{r}\right\}$ denote the sets of all longitudinal coordinates belonging to trajectories $C_{i}$ and $R_{j}$. The overlapping region $Y_{overlap}$ and the overlapping length $L_{overlap}$ of the two trajectories are defined as follows:

(1) $$Y_{overlap}\;=\;\left[\max\left(\min\left(Y_{C_{i}}\right),\min\left(Y_{R_{j}}\right)\right),\min\left(\max\left(Y_{C_{i}}\right),\max\left(Y_{R_{j}}\right)\right)\right],$$

(2) $$L_{overlap}=min\left(max\left(Y_{C_{i}}\right),max\left(Y_{R_{j}}\right)\right)-max\left(min\left(Y_{C_{i}}\right),min\left(Y_{R_{j}}\right)\right)$$

When the overlap length satisfies $L_{overlap}\geq L_{min}$, the sequences $Y_{C_{i}}^{overlap}$ and $Y_{R_{j}}^{overlap}$ within the overlapping interval are extracted:

(3) $$Y_{C_{i}}^{overlap}\;=\;\left\{y_{q}^{c}|y_{q}^{c}\in Y_{overlap},q\;=\;1,\ldots ,m\right\}\;=\;\left\{a_{1},a_{2},\ldots ,a_{M}\right\}$$

(4) $$Y_{R_{j}}^{overlap}\;=\;\left\{y_{l}^{r}|y_{l}^{r}\in Y_{overlap},l\;=\;1,\ldots ,n\right\}\;=\;\left\{b_{1},b_{2},\ldots ,b_{N}\right\}$$

where *M* and *N* denote the total number of data points retained within the overlapping region for the camera and radar trajectories, respectively. The sets $\left\{a_{1},a_{2},\ldots ,a_{M}\right\}$ and $\left\{b_{1},b_{2},\ldots ,b_{N}\right\}$ represent the newly formed longitudinal sequences that will be utilized as inputs for the subsequent DTW alignment process.

Next, we compute the DTW distance $D_{DTW}\left(A,B\right)$, which reflects the similarity in shape between two trajectories. A smaller distance indicates a higher likelihood that they originate from the same target. For two sequences $A\;=\;\{a_{1},a_{2},\ldots ,a_{M}\}$ and $B\;=\;\{b_{1},b_{2},\ldots ,b_{N}\}$ of lengths *M* and *N*, respectively, the DTW distance is computed via dynamic programming. Its recursive formulas are

(5) $$D_{DTW}\left(A,B\right)\;=\;\sqrt{D(A,B)}$$

(6) $$D\left(A,B\right)\;=\;d\left(a_{u},b_{v}\right)+\min\left\{D\left(a_{u-1},b_{v}\right),D\left(a_{u},b_{v-1}\right),D\left(a_{u-1},b_{v-1}\right)\right\}$$

(7) $$d\left(a_{u},b_{v}\right)\;=\;{\left(a_{u}-b_{v}\right)}^{2}$$

where *u* and *v* denote the indices for sequences *A* and *B*, $d(a_{u},b_{v})$ denotes the distance metric between trajectory points, and D(A,B) denotes the cumulative distance matrix. The optimal path is obtained by calculating D(A,B).

We construct the cost matrix *S* in the Hungarian matching algorithm using the computed DTW distances:

(8) $$S\left(C_{i},R_{j}\right)\;=\;D_{DTW}\left(Y_{C_{i}}^{overlap},Y_{R_{j}}^{overlap}\right)$$

If two trajectories lack sufficient overlap, set $S(C_{i},R_{j})\;=\;\infty$. Based on the cost matrix *S*, the Hungarian algorithm is employed to solve for the optimal one-to-one correspondence matching [34], ensuring each camera trajectory matches at most one radar trajectory while minimizing the global matching cost.

#### 3.3.2. Spatio-Temporal Parameter Estimation

For successfully matched trajectory pairs, the alignment paths generated by the DTW algorithm establish spatio-temporal correspondences between the trajectory points of the two sensors. For each trajectory *k*, the time offset $\Delta t_{k}$ is calculated using

(9) $$\Delta t_{k}=t_{C_{k}}-t_{R_{k}}$$

and the sliding window differential method is employed to estimate the instantaneous velocity $v_{k}$:

(10) $$v_{k}\;=\;\frac{|y_{p_{k}}^{c}-y_{p_{k-N}}^{c}|}{t_{p_{k}}^{c}-t_{p_{k-N}}^{c}}$$

where *N* denotes the sliding window size and $p_{k}$ denotes the index in the trajectory sequence. The computed $v_{k}$ values are filtered to remove those with velocities approaching zero and non-finite values caused by measurement interruptions. After validity screening, the regression dataset is constructed.

Since the spatial coordinates of the camera and radar remain inconsistent, the time stamp deviation between their measurements of the same target can be regarded as a combined result of temporal and spatial offsets, as follows:

(11) $$\Delta t_{k}=\Delta T+\frac{\Delta Y}{v_{k}}+e_{k}$$

where ΔT represents the temporal offset, ΔY denotes the spatial offset, and $e_{k}$ is the measurement noise. Let $x_{k}=\frac{1}{v_{k}}$ and $y_{k}=\Delta t_{k}$; then, the regression model can be expressed as

(12) $$y_{k}=\Delta T+\Delta Y\times x_{k}+e_{k}$$

After collecting all valid alignment point data, let $\beta ={\left[\Delta T,\Delta Y\right]}^{T}$, and estimate the parameters ΔT and ΔY using least squares regression:

(13) $$\beta \;=\;{\left(X^{T}X\right)}^{-1}X^{T}Y$$

(14) $$X\;=\;\left[\begin{array}{cc} 1 & x_{1}\\ 1 & x_{2}\\ \vdots & \vdots \\ 1 & x_{M} \end{array}\right]$$

(15) $$Y\;=\;\left[\begin{array}{c} y_{1}\\ y_{2}\\ \vdots \\ y_{M} \end{array}\right]$$

Using the above method, we estimated the temporal offset ΔT and spatial offset ΔY, which served as the initial values for the subsequent spatio-temporal synchronization optimization model.

### 3.4. Spatio-Temporal Synchronization Optimization Model

Since the world coordinate system and radar coordinate system obtained after the preliminary calibration do not fully match, we adopted the spatio-temporal synchronization model $\left(\Delta T,\Delta X,\Delta Y,\theta ,K_{x},K_{y},{\left(e_{x-i},e_{y-i}\right)}_{i=1}^{3}\right)$ established by DU [15] and correct the world coordinates using the following equation:

(16) $$\left[K_{x}\;K_{y}\right]\left[\begin{array}{cc} \cos\theta & -\sin\theta \\ \sin\theta & \cos\theta \end{array}\right]\left[\begin{array}{c} x_{w-i}+e_{x-i}\\ y_{w-i}+e_{y-i} \end{array}\right]+\left[\begin{array}{c} \Delta X\\ \Delta Y \end{array}\right]=\left[\begin{array}{c} x_{wr-i}\\ y_{wr-i} \end{array}\right]$$

where $K_{x}$ and $K_{y}$ represent the scaling factors for the x- and y-axes of the two coordinate systems, respectively; θ denotes the planar angular deviation; $\left(x_{w-i},y_{w-i}\right)$ indicates the world coordinates of the *i*-th point, while $\left(e_{x-i},e_{y-i}\right)$ denotes the selection error; and $\left(x_{wr-i},y_{wr-i}\right)$ denotes the coordinates of the *i*-th point corrected from the world coordinate system to the radar coordinate system.

We utilize the previously calculated temporal offset ΔT to calibrate the camera time and compute the Euclidean distance of the vehicle trajectory as the objective function *F*, as follows:

(17) $$F\;=\;\min\left(\mathrm{mean}{\left(\mathrm{media}\times {\left(\sqrt{{\left(x_{wr-i}-x_{r-i}\right)}^{2}+{\left(y_{wr-i}-y_{r-i}\right)}^{2}}\right)}_{i=1}^{n}\right)}_{j=1}^{k}\right)$$

where $\left(x_{r-i},y_{r-i}\right)$ represents the radar coordinates of the *i*-th point, *n* denotes the number of frames during the public detection period, and *k* denotes the total number of targets. By iteratively adjusting the values of each parameter to minimize the objective function, we obtain the optimal solutions for all parameters.

## 4. Experiments and Analysis

### 4.1. Experimental Setup and Environment

In the context of this study, we provide a quantitative operational definition for “sparse scenarios” based on the limitations of the baseline method, which relies on feature vectors constructed from the time headways of N preceding and following neighbors (typically N = 3) [15]. Specifically, a traffic sequence is classified as sparse if the total number of targets (K) within the observation window is insufficient to populate the feature vector (i.e., K < 2N + 1, or K < 7 in our setup). Under these conditions, feature degeneration occurs due to zero-padding, leading to matching ambiguity.

**Figure 2:**
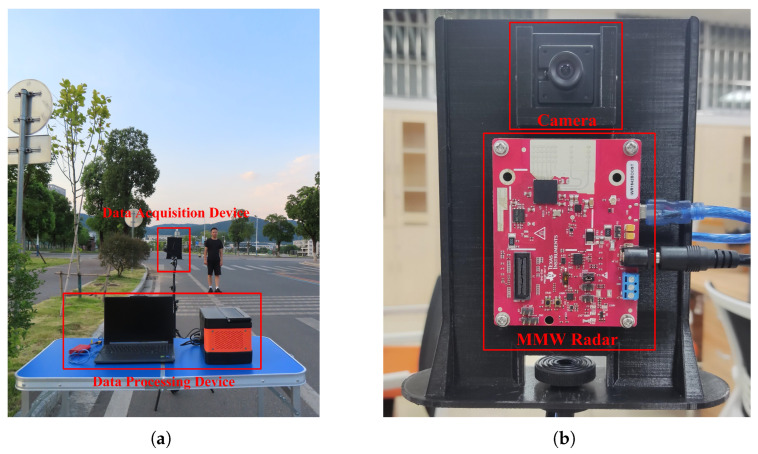
Experimental platform and equipment: (**a**) data processing device; (**b**) data acquisition device.

To validate the performance of the proposed method in sparse traffic environments, the experimental platform and equipment constructed in this paper are shown in Figure 2. It primarily consists of two components: a data processing unit and a data acquisition unit.

The data processing device (Figure 2a) receives raw data from the data acquisition unit, executes target detection and tracking algorithms, and implements multi-sensor data fusion. The data acquisition device (Figure 2b) comprises an IWR 1642 mm-wave radar (Texas Instruments, Dallas, TX, USA) and a high-definition USB camera (Shenzhen Blue Sky Technology Co., Ltd., Shenzhen, China).It communicates with the data processing unit via a serial bus for data exchange. Sensor parameter configurations are shown in Table 1. In the experiments, we collected a limited amount of pedestrian data to demonstrate the unsuitability of the DU method for sparse traffic scenarios and to validate the feasibility of our proposed approach.

While vehicles are the primary targets in Vehicle-to-Everything (V2X) systems, we deliberately selected pedestrian data to validate the proposed synchronization algorithm. Pedestrians, as critical Vulnerable Road Users (VRUs), present a significantly higher challenge for spatio-temporal synchronization compared with vehicles. Inherently, a vehicle is a rigid body characterized by a large Radar Cross-Section (RCS) and smooth, predictable kinematic constraints. In contrast, a pedestrian is non-rigid, possesses a small RCS, and exhibits highly irregular and highly maneuverable motion patterns. By successfully solving the spatio-temporal synchronization problem on these challenging pedestrian trajectories, we demonstrate the effectiveness of the proposed DTW-based method.

### 4.2. Data Preprocessing

**Figure 3:**
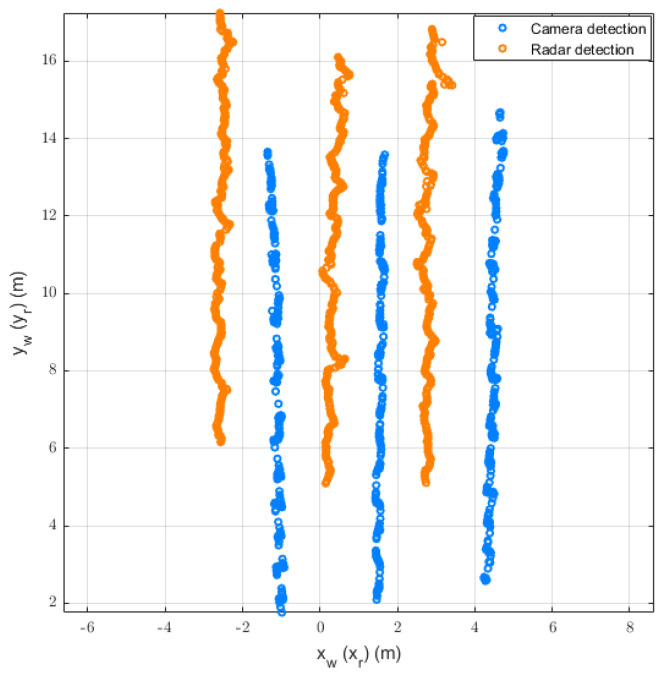
World and radar coordinates of the objects.

After acquiring data from the radar and camera, we performed linear interpolation on the radar data and processed the video using the DetectoRS detection algorithm to obtain the target’s bounding box. Subsequently, the pixel coordinates were transformed into world coordinates using the homography matrix calculated from prior road information. Figure 3 illustrates the radar coordinates and world coordinates after preliminary calibration of the objects, where the world coordinate system established through preliminary calibration still exhibits discrepancies relative to the radar coordinate system.

### 4.3. Analysis of Spatio-Temporal Synchronization Results

Based on the established spatio-temporal synchronization model, we randomly sampled 100 sets of initialization vectors within the feasible parameter range. By running the constrained nonlinear minimization solver in parallel, we ultimately selected the solution set with the smallest residuals for the objective function as the global optimal estimate (Table 2). The optimization model converged to a physically plausible solution. The temporal residual ΔT = 0.116 s indicates precise fine-tuning following the coarse linear regression. Spatially, the scale factors ($K_{y}$ ≈ 1.0) and rotation angle (θ ≈ 0°) confirm the high degree of consistency of the coordinate systems along the primary motion direction. Regarding the lateral scale factor, the value $K_{x}$ = 0.811 reflects a necessary scaling compensation. This deviation from unity is attributed to the inherent lateral perspective distortion of the monocular camera and the slight anisotropy in the radar’s azimuth range resolution. By adaptively adjusting $K_{x}$, the model effectively aligns the disparate lateral measurements. Furthermore, the translation parameters (ΔX,ΔY) accurately reflect the sensor installation offsets. Notably, the model adaptively identified significant errors in the first control point ($e_{x-1}$,$e_{y-1}$), effectively compensating for the perspective distortion at the far end of the field of view.

**Figure 4:**
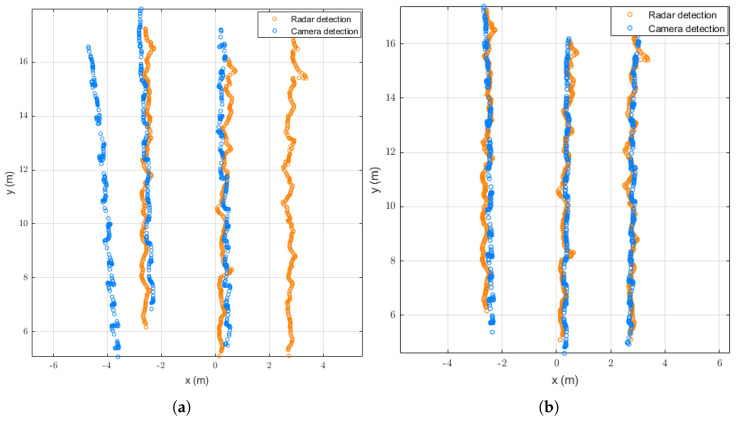
Trajectory comparison after spatio-temporal synchronization: (**a**) DU et al.’s method; (**b**) our proposed method.

Figure 4 shows a comparison of trajectories after spatio-temporal synchronization for the trajectories in Figure 3, using DU et al.’s method and our proposed method. Figure 4a shows that the virtual detection line approach proposed by DU suffers from target matching errors in scenarios with sparse target traffic. Specifically, the two trajectories on the left side of the radar are incorrectly matched with the two trajectories on the right side of the camera, while the far-right radar trajectory is matched with the far-left camera trajectory. During spatio-temporal synchronization, the two sequentially transformed trajectories were prioritized, making the mismatched pair appear well-synchronized. Figure 4b demonstrates that our proposed method achieves accurate target matching and spatio-temporal synchronization, even in sparse scenarios.

**Figure 5:**
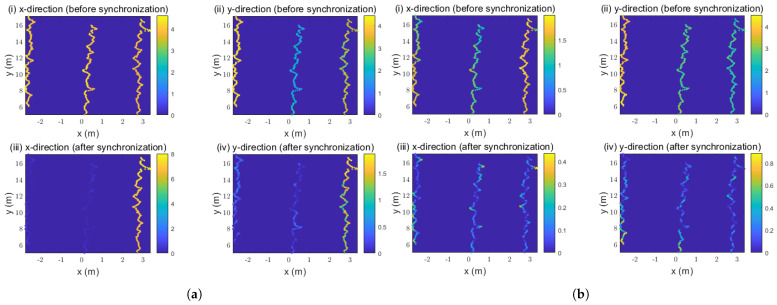
Comparison of spatial deviations before and after synchronization: (**a**) DU et al.’s method; (**b**) our proposed method; (**i**) x-direction (before synchronization); (**ii**) y-direction (before synchronization); (**iii**) x-direction (after synchronization); (**iv**) y-direction (after synchronization).

To further validate the effectiveness of our proposed method, we conducted a comparative analysis of the two approaches in the spatial and temporal dimensions. Figure 5 illustrates the spatial distribution comparison of trajectory deviations between the two methods, with the lines representing the target’s trajectory.

Figure 5a shows that the method proposed by DU exhibits significant misalignment during the target-matching phase, causing a substantial impact on the matched trajectories in the x-direction. It can be seen that the deviation of two misaligned trajectories has decreased, while the deviation of the other has increased. Overall, the average x-direction deviation decreased from 4.0508 to 2.6265 m, while the average y-direction deviation decreased from 3.0732 to 0.4415 m. Figure 5b shows that after successful target matching, our method significantly reduces the trajectory deviation in the x- and y-directions for each trajectory. The average deviation in the x-direction decreased from 1.4358 to 0.1074 m, while the average deviation in the y-direction decreased from 3.0732 to 0.1775 m.

**Figure 6:**
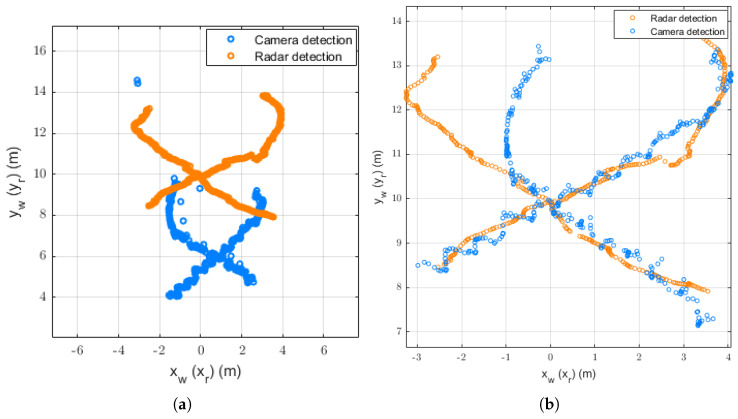
Trajectory comparison before and after spatio-temporal synchronization: (**a**) before synchronization; (**b**) after synchronization.

To validate temporal synchronization, we calculated residuals in the velocity curves using a set of pedestrian crossing data (Figure 6). Since pedestrians may exhibit negligible velocity changes in the y-direction, we employed lane changes in the x-direction to assess the temporal synchronization feasibility of our proposed method.

Figure 6 visualizes the synchronization performance in a challenging “two-person crossing” scenario. Before synchronization, the trajectories from the camera and radar exhibit significant spatial misalignment and shape distortion due to coordinate system disparity and time latency. After applying the proposed spatio-temporal synchronization, the two modalities show a high degree of alignment. Crucially, the method demonstrates superior robustness in the trajectory intersection area. This confirms that the proposed DTW-based matching strategy effectively leverages global geometric features to maintain correct data association, even in complex interaction scenarios, and the calibrated spatial parameters successfully unify the heterogeneous coordinate systems.

**Figure 7:**
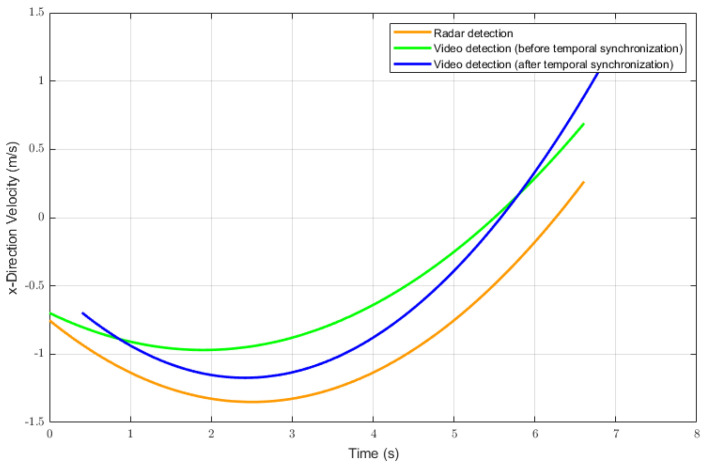
Time–velocity curves before and after temporal synchronization.

We plotted the x-direction velocities of targets detected by the video and radar and calculated the corresponding residuals (Figure 7). Visually, the synchronization effect is particularly evident in the phase alignment. The velocity troughs of the video curve, which originally led the radar curve, have shifted to align with the radar curve’s troughs after the correction. This demonstrates the effectiveness of our proposed method in achieving temporal synchronization. Additionally, the mean residual was 0.3891 m/s prior to the temporal synchronization and decreased to 0.2390 m/s following the synchronization. Since pedestrian motion involves non-rigid deformation and high maneuverability—inherently lacking the smoothness and predictability of vehicular rigid-body dynamics—the calculated residuals are not as low as those typically observed for vehicles.

### 4.4. Quantitative Evaluation of Matching Accuracy

Although the visual qualitative demonstration of spatio-temporal synchronization results reveals the baseline method’s susceptibility to mismatch errors, a comprehensive quantitative assessment is necessary to validate the robustness of the proposed data association strategy. To address this, we conducted a systematic evaluation of target matching accuracy across different traffic density levels. We manually annotated the ground truth correspondences for a subset of the dataset, categorizing the scenarios based on the number of targets (K). The matching accuracy is defined as the ratio of correctly paired radar–camera trajectories to the total number of ground truth pairs.

To systematically evaluate the matching performance across different density levels, we stratified the collected dataset into three distinct groups based on the total number of targets. For each group, we conducted three or four independent experimental trials to ensure the statistical reliability of the results (Table 3).

Table 3 shows that, under sparse flow conditions, the proposed method outperforms the baseline method regarding the matching accuracy. In sparse scenarios (K≤4), our method maintains a high accuracy, effectively resolving the feature degeneration problem. In denser scenarios (*K* = 5–6), although both methods suffer from occlusion and clutter, the proposed method still shows a clear advantage by leveraging the trajectory geometry. It should be noted that when K = 1, although the feature vectors of the baseline are all 0, the Hungarian algorithm is forced into a one-to-one matching and can still achieve correct matching. However, due to the degeneracy of the feature vectors, the synchronization parameters derived from the matching become invalid and divergent.

### 4.5. Computational Efficiency Analysis

From an algorithmic perspective, the baseline method relies on an iterative grid search strategy. Let *L* denote the number of trajectory points and *K* denote the number of targets. To estimate the spatial offset ΔY, the baseline method must scan the parameter space with *S* steps (e.g., S=40 for a 20 m range with 0.5 m increments), executing feature extraction (O(K·L)) and Hungarian matching $\left(O\left(K^{3}\right)\right)$ at every step. This results in a total complexity of $O(S\cdot \left(K^{3}+K\cdot L\right))$, where the search multiplier S dominates the computational load. In contrast, the proposed method avoids this iterative search entirely. By deriving ΔY analytically via linear regression after a one-time DTW alignment, we achieve a closed-form solution with a complexity of $O(K^{2}L^{2}+K^{3})$.

Furthermore, the implementation efficiency differs significantly. The baseline method incurs substantial overhead from frequent memory allocation and context switching due to the repetitive execution of interpolation and matching functions within the search loop. Conversely, the DTW calculation, despite its higher theoretical operation count, is based on dense matrix operations that are highly amenable to vectorization. This allows our method to fully leverage the Single Instruction, Multiple Data (SIMD) parallel acceleration capabilities of modern CPUs, drastically reducing the execution time per operation.

To validate this analysis, we recorded the average runtime on our experimental dataset. The results demonstrate a significant efficiency advantage: the proposed method runs in just 0.1058 s, while the baseline method requires 0.6016 s.

## 5. Conclusions

To address the limitation of existing radar–camera synchronization models, which heavily rely on dense traffic flow, we propose a spatio-temporal parameter estimation method based on DTW. Specifically, the DTW algorithm is employed to extract the global geometric similarity of asynchronous trajectories, establishing high-confidence, non-linear alignment paths between heterogeneous sensors, even in highly sparse scenarios. Subsequently, by exploiting the kinematic constraints inherent in the aligned trajectory points, a least squares linear regression model is constructed. This model analytically decouples the complex matching errors into the temporal offset and spatial deviation, eliminating the need for exhaustive spatial search iterations. Our proposed method achieves reliable spatio-temporal synchronization using only a small number of target trajectories, thereby significantly improving the computational efficiency while ensuring the accuracy of target matching. The method’s effectiveness and feasibility were validated in the experiment by collecting a small amount of pedestrian data. The results demonstrated that the average deviation in the x-direction decreased from 1.4358 to 0.1074 m, while the average deviation in the y-direction decreased from 3.0732 to 0.1775 m. Furthermore, the time synchronization effectiveness of this method was verified using cross-validation with two sets of data. Moreover, comparative evaluations of the matching accuracy indicated that the proposed approach consistently outperformed the baseline method regarding the target matching accuracy, successfully mitigating the misassociation issues in sparse traffic conditions. Additionally, by replacing the iterative space search with the method we propose, the execution time was significantly reduced. Consequently, this study provides an effective algorithmic foundation for the spatio-temporal synchronization of roadside perception units. In the future, we will consider further optimizing this algorithm to ensure it operates efficiently, even in environments with a high target traffic density.

## Figures and Tables

**Table 1:** Sensor parameters.

MMW Radar Parameters	Value (Unit)	Camera Parameters	Value (Unit)
Maximum detection range	56.2485 m	CMOS sensor model	IMX335
Maximum speed	7.7950 m/s	Resolution	640 × 480
Distance resolution	0.5 m	Focal length	2.8 mm
Speed resolution	0.1247 m/s	Field of view	95°
Sampling frequency	30 Hz	Sampling frequency	30 Hz

**Table 2:** Parameter results of the spatio-temporal optimization model.

Parameter	Lower Boundary	Upper Boundary	Result
ΔT (s)	0	1	0.116
ΔX (m)	−5	5	−0.842
ΔY (m)	−5	5	2.719
θ (°)	−1	1	−0.007
$K_{x}$	0.5	1.5	0.811
$K_{y}$	0.5	1.5	1.002
$e_{x-1}$ (m)	−1	1	−0.969
$e_{y-1}$ (m)	−1	1	0.893
$e_{x-2}$ (m)	−1	1	−0.027
$e_{y-2}$ (m)	−1	1	−0.383
$e_{x-3}$ (m)	−1	1	−0.148
$e_{y-3}$ (m)	−1	1	−0.173

**Table 3:** Quantitative comparison of matching accuracy.

Number of Targets (K)	Baseline Accuracy	Proposed Accuracy
1–2	71.4%	100%
3–4	61.5%	84.6%
5–6	36.4%	54.5%

## Data Availability

The raw data supporting the conclusions of this article will be made available by the authors upon request.
